# Risk of tuberculosis among air passengers estimated by interferon gamma release assay: survey of contact investigations, Japan, 2012 to 2015

**DOI:** 10.2807/1560-7917.ES.2017.22.12.30492

**Published:** 2017-03-23

**Authors:** Masaki Ota, Seiya Kato

**Affiliations:** 1 Research Institute of Tuberculosis, Japan Anti-Tuberculosis Association, Matsuyama 3-1-24, Kiyose city, Tokyo, Japan

**Keywords:** air-borne infections, tuberculosis, epidemiology, outbreaks, air travel, interferon gamma release assay

## Abstract

Although the World Health Organization recommends contact investigations around air travel-associated sputum smear-positive tuberculosis (TB) patients, evidence suggests that the information thus obtained may have overestimated the risk of TB infection because it involved some contacts born in countries with high TB burden who were likely to have been infected with TB in the past, or because tuberculin skin tests were used, which are less specific than the interferon gamma release assay (IGRA) particularly in areas where Bacillus Calmette-Guérin (BCG) vaccination coverage is high. We conducted a questionnaire survey on air travel-associated TB contact investigations in local health offices of Japan from 2012 to 2015, focusing on IGRA positivity. Among 651 air travel-associated TB contacts, average positivity was 3.8% (95% confidence interval (CI): 2.5–5.6) with a statistically significant increasing trend with older age (p < 0.0094). Positivity among 0–34 year-old contacts was 1.0% (95% CI: 0.12–3.5%), suggesting their risk of TB infection is as small as among Japanese young adults with low risk of TB infection (positivity: 0.85–0.90%). Limiting the contact investigation to fewer passengers (within two seats surrounding the index case, rather than two rows) seems reasonable in the case of aircraft with many seats per row.

## Introduction

International air travel has become widely accessible and the International Civil Aviation Organization has forecast that scheduled passenger traffic around the world will more than double, from 2.7 billion in 2011 to 6 billion annually by 2030 [1]. This will increase the frequency of transmission of communicable diseases [2] such as influenza [3], measles [4], SARS [5] and particularly tuberculosis (TB) during air travel [6].

The World Health Organization (WHO) issued a guideline on TB and air travel in 1998, and the third edition was published in 2008 [7], recommending that member states should conduct contact investigations for close contacts of not only smear-positive but also culture-positive TB patients, if the index case was diagnosed with multidrug-resistant TB. However, the guideline itself acknowledges that the available evidence for the risk of TB transmission during air travel and outcome data from passenger contact investigations are limited and it calls for a coordinated international approach to research, data collection, analysis and dissemination to strengthen the evidence base for operational decision-making and policy development. Moreover, a systematic review on contact investigations associated with air travel in 2010 argued that the evidence for TB transmission in commercial aircraft is limited and that there is reason doubt the value of actively screening air passengers for infection with *Mycobacterium tuberculosis* [8]. A more recent systematic review on the subject did not find any further evidence of TB transmission and concluded that the risk of TB transmission aboard aircraft seems to be very low [9].

The challenges in estimating risk of contracting TB infection associated with air travel include the difficulty of obtaining the appropriate evidence: (i) Contact investigation for air passengers is often complicated by the unavailability or reluctance of the airline companies to share the flight manifest and by the unavailability of contacts. (ii) Contacts may have been infected with TB in the past, e.g. those born in countries with a high burden of TB. (iii) The specificity of tuberculin skin testing (TST) used in most contact investigations is low, leading to high positivity among the contacts, e.g. 24% in data from the United States Centers for Disease Control and Prevention (US CDC) [10].

In Japan, the TB notification rate has declined in the past six decades from 698.4 per 100,000 population in 1951 to 17.7 per 100,000 population in 2013 [11], which is equivalent to the rate in Poland (17.6/100,000) and Estonia (18.4/100,000) in 2014 [12]. However, 8,000 smear-positive TB cases are still reported every year [13] and more than 65% of those involve persons aged 65 years or older, reflecting the ageing population. Therefore, incidents in which infectious, particularly elderly, TB cases travel by air unaware of their infectiousness, are not uncommon. On the other hand, almost all children and young adults are estimated to be uninfected [14-16], therefore the positivity among children and young adults could be used as a surrogate marker for the risk of contracting TB in contact investigations. The local governments of Japan usually comply with the WHO guidelines and conduct contact investigations for contacts of smear-positive index TB patients associated with air travel. However, in Japan, no literature has been published on the contact investigations associated with air travel and the outcomes of contact investigations have not been reported.

Interferon gamma release assays (IGRA) can diagnose latent TB infection more sensitively and specifically than TST because TST also reacts to Bacillus Calmette-Guérin (BCG) vaccination and the interpretation of TST results is likely to be ambiguous where BCG vaccination coverage is high [15-18]. Two IGRA are currently available in Japan, the T-Spot TB (T-SPOT; Oxford Immunotec, Abingdon, United Kingdom) and the QuantiFERON-TB Gold In-Tube assay (QFT-GIT, Qiagen, the Netherlands), and they are widely used in contact investigations, including those associated with air travel [19].

We conducted a questionnaire survey on air travel-associated TB contact investigations in the local health offices of Japan from 2012 to 2015, focusing on IGRA positivity among the contacts. The purpose of the study was to estimate the risk of TB transmission associated with air travel, particularly using the IGRA positivity among children and young adult contacts as the outcome indicator.

## Methods

### Case definition for contact investigation associated with air travel

An incident of infectious TB involving air travel was defined as an event in which the WHO guidelines for initiating contact investigations were met [7] and in which the local health offices decided to undertake an investigation. Events with an index case with smear-negative TB or unknown smear status, or with a flight duration shorter than 6 hours were excluded. Since in most of the contact investigations, only the flight time was available but not the ground delays after boarding or after landing, we decided that a flight duration of 6 hours or more would meet the definition of the total flight duration of 8 hours or longer stipulated in the WHO guideline.

### Contact investigations of tuberculosis contacts in Japan

The practice of contact investigations of TB contacts in Japan is similar to that recommended elsewhere [20]. Briefly, once a TB case is reported to a local health office by a physician, a public health nurse of the health office where the patient lives visits the patient to conduct an interview about contacts. When the case is smear-positive, the health office initiates a contact investigation (initiator health office). When a contact is a resident of another health office's jurisdiction, the initiator health office requests the health office at the residency (implementer health office) to conduct health screening for the contact on its behalf. When a ministry of health of a foreign country requests the national TB programme (NTP) of Japan to conduct a contact investigation associated with air travel for a Japanese resident, the NTP asks the health office of the contact's residence to conduct health screening. The health screening usually involves IGRA tests and, if indicated, a chest X-ray.

In contact investigations associated with air travel, the initiator health office usually obtains information from the airline company on seating positions of the index case as well as the contacts who were seated in the two rows in front of and behind the index case and on the contact details of the contacts. It then sends letters to the health offices where the contacts live to request health screening. When a contact is a resident of a foreign country, the initiator health office normally asks the NTP of Japan to coordinate the investigation with the ministry of health of that country.

### Data collection

In November 2015, we sent a questionnaire to all 486 local health offices in Japan and asked whether they had conducted contact investigations associated with air travel from 2012 through October 2015. Those who conducted contact investigations as either an initiator or an implementer health office, or both, were further asked about the index cases and the outcomes of the contact investigations via a structured questionnaire. The data collection was conducted from late November 2015 through March 2016.

Data collected included characteristics of the index case (age group, sex, smear test result, presence of cough at diagnosis and a brief description of chest X-ray shadow), the boarded flights (flight numbers, destinations and duration), outcomes of the contact investigation, particularly the number of the eligible contacts defined as those who were on two rows in front of and behind the index case, the number of contacts screened for TB, including the number of the contacts with IGRA, and how many were positive in the IGRA. Those contacts who also had contact with the index case outside the airplane, such as family members or travel companions, were excluded.

### Data entry

The data on the events were entered into Microsoft Excel. The events reported both from the initiator and the implementer health offices were sorted by the date of the flight, the flight number or the airline company, and the destination. When we found duplicated events, only the data reported from the initiator health offices were used. The events with unknown flight dates, unknown flight numbers or airline companies or unknown destinations were excluded.

### Data analysis

The investigated contacts were pooled and classified by age groups, and the positivity was calculated as a whole, by age under 35 years and by age groups.

### Statistical tests

A binomial estimation of the 95% confidence intervals (CI) was performed using R software (Version 3.01, The R Foundation for Statistical Computing, Vienna, Austria) to compare the IGRA positivity between the age groups.

## Results

Of the 486 local health offices of Japan, 451 (93%) responded. Table 1 shows the overview of the questionnaire survey. A total of 17 health offices reported that they took the lead in one or more of the contact investigations on 19 index TB patients who boarded airplanes between February 2012 and September 2015. The median duration between the dates of the air travel and the TB diagnosis of the patients was 1 month, ranging from 1 to 4 months. The total number of eligible contacts the initiator health offices reported, excluding those who had contact with the index cases outside of the airplanes, was 942, of whom 574 (61%) were screened for TB and 523 (56%) had IGRA test results available. Six eligible contacts declined TB screening. Thus, the response rate (the sum of those who were screened and who declined, divided by the number of eligible contacts) was 62%. An additional 70 health offices reported that they implemented the contact investigations for one or more of the contacts of 23 index TB patients (requested by foreign countries and the health offices that did not respond in our study) and provided IGRA test results on 128 contacts.

**Table 1:** Results of questionnaire survey on tuberculosis contact investigations among air passengers, Japan, 2012–2015 (n = 651 IGRA-tested)

Reporting health offices	Initiator health offices^a^	Implementer health offices^b^
Number of health offices reported	17	70
Number of index TB cases	19	23
Number of flights involved in contact investigations	35	27
Median duration of flights in hours (range)	11 (6–12)	10 (7–12)
Number of eligible contacts^c^	942 (100%)	unknown
Number of eligible contacts reached^d^	580 (61.6%)	unknown
Number of eligible contacts screened for TB	574 (60.9%)	unknown
Number of eligible contacts tested with IGRA	523 (55.5%)	128

IGRA: interferon gamma release assay; TB: tuberculosis.

^a^ Initiator health office: the health office that initiated the contact investigation.

^b^ Implementer health office: the health office that implemented health screening for the contacts at the request of the initiator health office.

^c^ Those contacts who had contact with the index cases outside the aircrafts were excluded.

^d^ Number of eligible contacts reached is a sum of the number of eligible contacts screened and the number of eligible contacts who declined being tested.

Of the total 651 contacts, 25 (3.8%; 95% CI: 2.5–5.6) were positive for IGRA (Table 2). Among 205 contacts aged 0–34 years, two (1.0%; 95% CI: 0.12–3.5) were positive for IGRA. All of the 651 contacts were resident in Japan, however, details on their nationality were not known. The Cochran–Armitage test revealed that there was a statistically significant increasing trend towards a correlation between age group and positivity of IGRA test results (p < 0.0094). 

**Table 2:** Positivity of interferon gamma release assay among tuberculosis contacts during air travel, by age groups, Japan, 2012–2015 (n = 651)

Age group (years)	Contacts investigated	IGRA-positive contacts	IGRA positivity (%)^a^	95% LCL	95% HCL
0–14	20	0	0.0	0.0	16.8
15–24	46	1	2.2	0.0	11.5
25–34	139	1	0.7	0.0	3.9
35–44	140	3	2.1	0.4	6.1
45–54	115	6	5.2	1.9	11.0
55–64	86	6	7.0	2.6	14.6
65–74	70	7	10.0	4.1	19.5
75–84	13	0	0.0	0.0	24.7
Unknown	22	1	4.5	0.1	24.7
**Total**	**651**	**25**	**3.8**	**2.5**	**5.6**

HCL: higher confidence limit; IGRA: interferon gamma release assay; LCL: lower confidence limit.

^a^ A Cochran-Armitage test revealed there was a statistically significant increasing trend between age group and positivity of IGRA test results (p < 0.0094).

For eight contacts with negative IGRA test results reported by the implementer health offices, the information on flight date, flight route or flight number was not available, and we were unable to cross-check this with the information from the initiator health offices. Thus, we excluded the eight contacts from the database. 

No contact developed TB disease after contact with a TB case on an airplane.

## Discussion

We conducted a questionnaire survey on air travel-associated TB contact investigations conducted in Japan. We found that 3.8% of the contacts had positive IGRA test results, with the positivity among the child and young adult contacts being 1.0%, which is almost equivalent to the IGRA positivity in Japanese medical students with no previous risk of TB infection (0.85%) [15] and in healthy university students (0.90%) [16]. This suggests that the risk of contracting TB infection associated with air travel is minuscule.

This level of risk is consistent with published data (0–4%) from 1993 to 2008 [10,21-23] but much lower than the risk reported in the early 1990s (30%) [24,25].

There was a statistically significant increasing trend of IGRA positivity with older age. This might reflect accumulated TB infection in the past [26,27], particularly in the 1950s and 1960s when the TB notification rates in Japan were higher than 150 per 100,000 population [28], rather than recent TB infection associated with air travel. Even in the late 1970s, the TB notification rates were higher than 60 per 100,000 population [28]. We therefore believe that it is reasonable to exclude those aged older than 35 years when analysing the risk of TB associated with air travel in our study.

The reason why the risk of TB infection associated with air travel is minuscule is that most commercial aircrafts used for long-distance flights have installed good ventilation systems with air exchange rates of more than 10 times per hour [29] and HEPA filters [30], which are equivalent to the requirements for isolation areas of healthcare facilities in the US [31], reducing the risk of TB infection during air travel. We have collected the IGRA test results of more than 600 passenger contacts, enabling us to stratify them into age groups and analyse the data of the age group of 0–34 years-olds, who are least likely to have been infected with TB before the relevant air travel.

In addition, since the positivity of IGRA was used as the main outcome indicator for the contact investigation, the data we report here were more sensitive and specific than those obtained using TST, particularly for areas where BCG vaccination coverage is very high. As we obtained information on contact investigations of TB associated with air travel from almost all the health offices of Japan and included in this study, we believe that these data are representative of the risk of contracting TB infection during air travel to and from Japan.

However, our study has some limitations: Since most health offices did not conduct the IGRA tests for the contacts immediately after the contact with TB cases, we were not able to calculate conversion rates. Considering the delay between contact with a TB case, diagnosis of the TB case and initiation and implementation of the contact investigation by different health offices, we believe it would be next to impossible to conduct the first IGRA tests within two or three weeks of contact with a TB case, and thus this limitation is practically unavoidable.

Although we assumed that contacts younger than 35 years were almost naïve to TB infection before the relevant air travel, this may not have been the case. Combined with the unavailability of the IGRA conversion rates mentioned above, we may have overestimated the TB risk associated with air travel. However, considering the low IGRA positivity (1.0%) among children and young adult contacts, we believe the main conclusion would not change.

Because the study was a questionnaire survey administered to the health offices of Japan, it has additional limitations. Some health offices may not have reported having conducted air travel-associated contact investigations and thus may not be listed in our database. However, because we employed an inventory method to collect information on the contact investigations from both the initiator and the implementer health offices, including the central NTP unit, we believe that we have done our best to obtain an almost complete picture on air travel-associated contact investigations conducted in Japan.

The information some implementer health offices provided was incomplete and therefore excluded from the database, leading to a possible bias. However, considering that only eight contacts were excluded and that all of them were negative in IGRA, the potential bias is small and the IGRA positivity may be overestimated, but not underestimated. Finally, it should also be noted that the authors do not know the quality of IGRA tests conducted for the contacts at each health office.

From our findings, we believe that the WHO could narrow the criteria for initiating air travel-associated contact investigations to, for example, only smear-positive TB, as is recommended by the European Centre for Disease Prevention and Control (ECDC) in the risk assessment guidelines for infectious diseases transmitted on aircraft (RAGIDA) related to TB [32]. As the ECDC guideline further recommends, the infectiousness of the index case, such as transmission to household members or other close contacts, should be considered before initiating air travel-associated contact investigations [9]. As modelling studies suggest, the risk of contracting TB infection on an aircraft varies from low to moderate and is highest in the rows closest to the index case [33]. Limiting the contact investigation to fewer passengers (within two seats surrounding the index case, rather than two rows) in the case of wide aircraft with many seats per row seems reasonable [9]. Countries with a high burden of TB should prioritise other, more important, activities [8].

## References

[1] International Civil Aviation Organization (ICAO). Global air transport outlook to 2030 and trends to 2040 (ICAO Cir 333). Montreal: ICAO; 2013.

[2] Mangili A Gendreau MA Transmission of infectious diseases during commercial air travel. Lancet. 2005;365(9463):989-96. 10.1016/S0140-6736(05)71089-8 15767002 PMC7134995

[3] Klontz KC Hynes NA Gunn RA Wilder MH Harmon MW Kendal AP An outbreak of influenza A/Taiwan/1/86 (H1N1) infections at a naval base and its association with airplane travel. Am J Epidemiol. 1989;129(2):341-8. 10.1093/oxfordjournals.aje.a115137 2912044

[4] Nelson K Marienau K Schembri C Redd S Measles transmission during air travel, United States, December 1, 2008-December 31, 2011. Travel Med Infect Dis. 2013;11(2):81-9. 10.1016/j.tmaid.2013.03.007 23562445

[5] Olsen SJ Chang HL Cheung TY Tang AFY Fisk TL Ooi SPL Transmission of the severe acute respiratory syndrome on aircraft. N Engl J Med. 2003;349(25):2416-22. 10.1056/NEJMoa031349 14681507

[6] McFarland JW Hickman C Osterholm M MacDonald KL Exposure to Mycobacterium tuberculosis during air travel. Lancet. 1993;342(8863):112-3. 10.1016/0140-6736(93)91311-9 8100876

[7] World Health Organization (WHO). Tuberculosis and air travel: guidelines for prevention and control. 3rd ed. Geneva: WHO; 2008. Available from: http://www.who.int/tb/publications/2008/9789241547505/en/23785743

[8] Abubakar I Tuberculosis and air travel: a systematic review and analysis of policy. Lancet Infect Dis. 2010;10(3):176-83. 10.1016/S1473-3099(10)70028-1 20185096

[9] Kotila SM Payne Hallström L Jansen N Helbling P Abubakar I Systematic review on tuberculosis transmission on aircraft and update of the European Centre for Disease Prevention and Control risk assessment guidelines for tuberculosis transmitted on aircraft (RAGIDA-TB). Euro Surveill. 2016;21(4):30114. 10.2807/1560-7917.ES.2016.21.4.30114 26848520

[10] Marienau KJ Burgess GW Cramer E Averhoff FM Buff AM Russell M Tuberculosis investigations associated with air travel: U. S. Centers for Disease Control and Prevention, January 2007-June 2008. Travel Med Infect Dis. 2010;8(2):104-12. 10.1016/j.tmaid.2010.02.003 20478518

[11] Katsuda N Hirosawa T Reyer JA Hamajima N Roles of public health centers (Hokenjo) in tuberculosis control in Japan. Katsuda N, Hirosawa T, Reyer JA, Hamajima N. Nagoya J Med Sci. 2015;77(1-2):19-28. 25797967 PMC4361504

[12] European Centre for Disease Prevention and Control (ECDC). Surveillance atlas of infectious diseases. Tuberculosis - All cases - Notification rate: 2014. Stockholm: ECDC. [Accessed: 15 Jul 2016]. Available from: http://ecdc.europa.eu/en/data-tools/atlas/Pages/atlas.aspx

[13] Tuberculosis Surveillance Center RIT JATA [Tuberculosis annual report 2013--(4). Tuberculosis treatment and treatment outcomes]. Kekkaku. 2015;90(7):595-604. 26630730

[14] Ohmori M Ishikawa N Yoshiyama T Uchimura K Aoki M Mori T Current epidemiological trend of tuberculosis in Japan. Int J Tuberc Lung Dis. 2002;6(5):415-23. 12019917

[15] Ogiwara T Kimura T Tokue Y Watanabe R Nara M Obuchi T Tuberculosis screening using a T-cell interferon-γ release assay in Japanese medical students and non-Japanese international students. Tohoku J Exp Med. 2013;230(2):87-91. 10.1620/tjem.230.87 23759899

[16] Higuchi K Sekiya Y Igari H Watanabe A Harada N Comparison of specificities between two interferon-gamma release assays in Japan. Int J Tuberc Lung Dis. 2012;16(9):1190-2. 10.5588/ijtld.11.0829 22748102

[17] Diel R Loddenkemper R Nienhaus A Evidence-based comparison of commercial interferon-gamma release assays for detecting active TB: a metaanalysis. Chest. 2010;137(4):952-68. 10.1378/chest.09-2350 20022968

[18] Sester M Sotgiu G Lange C Giehl C Girardi E Migliori GB Interferon-γ release assays for the diagnosis of active tuberculosis: a systematic review and meta-analysis. Eur Respir J. 2011;37(1):100-11. 10.1183/09031936.00114810 20847080

[19] Fujikawa A Fujii T Mimura S Takahashi R Sakai M Suzuki S Tuberculosis contact investigation using interferon-gamma release assay with chest x-ray and computed tomography. PLoS One. 2014;9(1):e85612. 10.1371/journal.pone.0085612 24454900 PMC3891819

[20] Centers for Disease Control and Prevention Guidelines for the investigation of contacts of persons with infectious tuberculosis; recommendations from the National Tuberculosis Controllers Association and CDC, and Guidelines for using the QuantiFERON-TB Gold test for detecting Mycobacterium tuberculosis infection, United States. MMWR Recomm Rep. 2005;54:1-37. 16357823

[21] Kornylo-Duong K Kim C Cramer EH Buff AM Rodriguez-Howell D Doyle J Three air travel-related contact investigations associated with infectious tuberculosis, 2007-2008. Travel Med Infect Dis. 2010;8(2):120-8. 10.1016/j.tmaid.2009.08.001 20478520

[22] Whitlock G Calder L Perry H A case of infectious tuberculosis on two long-haul aircraft flights: contact investigation. N Z Med J. 2001;114(1137):353-5. 11587303

[23] Miller MA Valway S Onorato IM Tuberculosis risk after exposure on airplanes. Tuber Lung Dis. 1996;77(5):414-9. 10.1016/S0962-8479(96)90113-6 8959144

[24] Kenyon TA Valway SE Ihle WW Onorato IM Castro KG Transmission of multidrug-resistant Mycobacterium tuberculosis during a long airplane flight. N Engl J Med. 1996;334(15):933-8. 10.1056/NEJM199604113341501 8596593

[25] Driver CR Valway SE Morgan WM Onorato IM Castro KG Transmission of Mycobacterium tuberculosis associated with air travel. JAMA. 1994;272(13):1031-5. 10.1001/jama.1994.03520130069035 8089885

[26] Mori T Harada N Higuchi K Sekiya Y Uchimura K Shimao T Waning of the specific interferon-gamma response after years of tuberculosis infection. Int J Tuberc Lung Dis. 2007;11(9):1021-5. 17705982

[27] Seto J Ahiko T [Effectiveness of interferon-gamma release assays in the tuberculosis contact investigation of elderly people]. Kekkaku. 2014;89(4):503-8. 24908811

[28] Mori T Recent trends in tuberculosis, Japan. Emerg Infect Dis. 2000;6(6):566-8. 10.3201/eid0606.000602 11076712 PMC2640925

[29] Hocking MB Passenger aircraft cabin air quality: trends, effects, societal costs, proposals. Chemosphere. 2000;41(4):603-15. 10.1016/S0045-6535(99)00537-8 10819229

[30] Dowdall NP Evans AD Thibeault C Air Travel and TB: an airline perspective. Travel Med Infect Dis. 2010;8(2):96-103. 10.1016/j.tmaid.2010.02.006 20478517

[31] Sehulster L Chinn RY CDC HICPAC Guidelines for environmental infection control in health-care facilities. Recommendations of CDC and the Healthcare Infection Control Practices Advisory Committee (HICPAC). MMWR Recomm Rep. 2003;52(RR-10):1-42. 12836624

[32] European Centre for Disease Prevention and Control (ECDC). Risk assessment guidelines for infectious diseases transmitted on aircraft (RAGIDA) - Tuberculosis. Stockholm: ECDC; 2014. Available from: http://ecdc.europa.eu/en/publications/_layouts/forms/Publication_DispForm.aspx?List=4f55ad51-4aed-4d32-b960-af70113dbb90&ID=1098

[33] Ko G Thompson KM Nardell EA Estimation of tuberculosis risk on a commercial airliner. Risk Anal. 2004;24(2):379-88. 10.1111/j.0272-4332.2004.00439.x 15078308

